# Cervicovaginal mucus barrier properties during pregnancy are impacted by the vaginal microbiome

**DOI:** 10.3389/fcimb.2023.1015625

**Published:** 2023-03-31

**Authors:** Hannah C. Zierden, Kevin DeLong, Fareeha Zulfiqar, Jairo Ortiz Ortiz, Victoria Laney, Sabrine Bensouda, Nicole Hernández, Thuy M. Hoang, Samuel K. Lai, Justin Hanes, Anne E. Burke, Laura M. Ensign

**Affiliations:** ^1^Center for Nanomedicine at the Wilmer Eye Institute, Johns Hopkins University School of Medicine, Baltimore, MD, United States; ^2^Department of Chemical & Biomolecular Engineering, Johns Hopkins University, Baltimore, MD, United States; ^3^Department of Ophthalmology, Wilmer Eye Institute, Johns Hopkins University School of Medicine, Baltimore, MD, United States; ^4^Department of Pharmacology and Molecular Sciences, Johns Hopkins University School of Medicine, Baltimore, MD, United States; ^5^Division of Pharmacoengineering and Molecular Pharmaceutics, Eshelman School of Pharmacy, University of North Carolina/North Carolina State University (UNC/NCSU) Joint Department of Biomedical Engineering, Department of Microbiology & Immunology, University of North Carolina-Chapel Hill, Chapel Hill, NC, United States; ^6^Department of Biomedical Engineering, Johns Hopkins University, Baltimore, MD, United States; ^7^Department of Gynecology and Obstetrics, Johns Hopkins University School of Medicine, Baltimore, MD, United States

**Keywords:** vaginal microbiome, drug delivery, mucus, pregnancy, preterm birth (PTB)

## Abstract

**Introduction:**

Mucus in the female reproductive tract acts as a barrier that traps and eliminates pathogens and foreign particles via steric and adhesive interactions. During pregnancy, mucus protects the uterine environment from ascension of pathogens and bacteria from the vagina into the uterus, a potential contributor to intrauterine inflammation and preterm birth. As recent work has demonstrated the benefit of vaginal drug delivery in treating women’s health indications, we sought to define the barrier properties of human cervicovaginal mucus (CVM) during pregnancy to inform the design of vaginally delivered therapeutics during pregnancy.

**Methods:**

CVM samples were self-collected by pregnant participants over the course of pregnancy, and barrier properties were quantified using multiple particle tracking. 16S rRNA gene sequencing was performed to analyze the composition of the vaginal microbiome.

**Results:**

Participant demographics differed between term delivery and preterm delivery cohorts, with Black or African American participants being significantly more likely to delivery prematurely. We observed that vaginal microbiota is most predictive of CVM barrier properties and of timing of parturition. Lactobacillus crispatus dominated CVM samples showed increased barrier properties compared to polymicrobial CVM samples.

**Discussion:**

This work informs our understanding of how infections occur during pregnancy, and directs the engineering of targeted drug treatments for indications during pregnancy.

## Introduction

The female reproductive tract is equipped with a cervicovaginal mucus (CVM) barrier which protects the underlying epithelium from foreign pathogens. As the first line of defense against infection, CVM is a complex mixture of mucin proteins, ions, lipids, cells, and bacteria (Cone, 2009). The mesh-like structure of CVM aids in sterically hindering large particulates, while the charged and hydrophobic regions of mucus facilitate adhesive barrier properties (Cone, 2009; Ensign et al., 2012). Mucus is continually secreted and cleared, eliminating materials that become trapped in the sticky gel from the body. Despite the benefits of CVM to reproductive health, it presents a challenge to effective local drug administration. Vaginal drug delivery is optimal for targeting the female reproductive tract, leading to increased drug concentrations in local tissues while decreasing off-target side effects. However, vaginally delivered therapeutics must be designed to overcome the steric and adhesive barrier properties of mucus. We previously engineered nano-sized formulations coated with a hydrophilic, net-neutral polymer to prevent preterm birth (PTB) in preclinical murine models of progesterone withdrawal and intrauterine inflammation with vaginal administration (Hoang et al., 2019; Zierden et al., 2021a). While these studies demonstrated the potential benefit of vaginally delivered therapies in obstetric indications, the barrier properties of human CVM during pregnancy have yet to be defined.

In addition to protecting the female reproductive tract, during pregnancy, CVM serves to protect the fetal compartment from ascending bacteria and inflammation. Indeed, a compromised CVM barrier has the potential to increase a woman’s risk of adverse pregnancy outcomes, including PTB, the leading cause of infant mortality and morbidity (CDC, 2019a; CDC, 2019b). It is estimated that 25% of PTBs are due to intrauterine infection and inflammation (Cunningham et al., 2018; Zierden et al., 2020). One potential source of intrauterine inflammation is ascension of bacteria from the vagina into the uterine environment (Malaeb and Dammann, 2009; Norwitz and Caughey, 2011). Unlike most microbial communities in the human body, what is considered an optimal vaginal ecosystem in reproductive aged women consists of a low level of bacterial diversity, typically dominated by *Lactobacillus* species (Ravel et al., 2011). Conversely, ~30% of women in the United States are affected by bacterial vaginosis (BV), a state of dysbiosis where the microbial community is dominated by anaerobic bacteria, including *Gardnerella vaginalis* (Eschenbach, 1993; Kroon et al., 2018; Sobel, 2018). BV is associated with a higher risk for PTB (DiGiulio et al., 2015; Callahan et al., 2017), as well as a variety of other adverse obstetric and gynecologic outcomes (Bukusi et al., 2006; Koumans et al., 2007; Atashili et al., 2008; Nelson et al., 2015). Sonographic short cervix, a key risk factor for PTB, has been associated with vaginal colonization by *G*. *vaginalis* (Hassan et al., 2006; Gerson et al., 2019), potentially due to inflammation and altered cervical epithelial cell function (Anton et al., 2018; Sierra et al., 2018). Furthermore, it has been described that samples of the cervical mucus plug from women at high-risk of PTB showed decreased barrier function to particles and peptides *ex vivo* (Critchfield et al., 2013). In the context of BV, the production of mucin-degrading enzymes by BV-associated bacteria may lead to impaired protection of the uterine compartment from microbial ascension (Howe et al., 1999; Wiggins et al., 2001; Cauci et al., 2008; Lewis et al., 2013). Thus, the complex interplay between CVM barrier function and the vaginal microbiota plays a key role in pregnancy and neonatal health.

Multiple particle tracking (MPT) is a technique that has enabled characterization of the structural and barrier function of various human mucus secretions, including CVM (Suh et al., 2005; Lai et al., 2007; Lai et al., 2010; Schuster et al., 2015). By tracking the spatial location of various sizes of fluorescently-labeled probe nanoparticles with either mucoadhesive (conventional particles, CP) or mucoinert (mucus-penetrating particles, MPP) surface characteristics over time, both adhesive and steric interactions between particles and the mucus mesh can be observed. We previously employed MPT to characterize the impact of the vaginal microbiota composition on CVM barrier function in a cohort of non-pregnant participants. We observed decreased CVM barrier function to HIV virions and CP in polymicrobial samples from women with and without BV symptoms compared to CVM from women with vaginal microbiota dominated by *Lactobacillus* spp (Nunn et al., 2015; Hoang et al., 2020). Further, there was no restoration of barrier function after antibiotic treatment for BV (Zierden et al., 2020). In a small cohort of pregnant participants with healthy vaginal microbiota, we observed that there was a minor decrease in nanoparticle mobility in their CVM samples, suggesting that there may be an impact of hormones on pore size (Hoang et al., 2019). Here, we utilized MPT to probe the barrier properties and pore sizes of CVM samples collected from 92 pregnant participants in Baltimore City as a function of both weeks of pregnancy and the vaginal microbiota. Our work here suggests that the vaginal microbiome is more predictive of CVM barrier properties during pregnancy than other physical characteristics of CVM or weeks of pregnancy. By understanding the relationship between the vaginal microbiome and the mucus barrier over the course of pregnancy, we can both elucidate the mechanistic contribution to PTB risk and further define design criteria for effective vaginal therapeutics for obstetric applications (Mitchell and Marrazzo, 2014; Anton et al., 2018; Fettweis et al., 2019; Zierden et al., 2021a).

## Materials and methods

### Materials

Instead Softcups^®^ were obtained from Evofem (San Diego, CA). Wiretrol^®^ disposable micropipets were obtained from Drummond Scientific Co. (Broomall, PA). ESwabs™ were obtained from BD (Franklin Lakes, NJ) and hCG urine test strips were obtained from Clinical Guard (Atlanta, GA). A pH microelectrode was obtained from Microelectrodes, Inc. (Bedford, NH). D/L-lactic acid assay kits were obtained from R-Biopharm (Darmstadt, Germany). V-PLEX plates and assay kits were obtained from Meso Scale Discovery (Rockville, MD). DNeasy^®^ Blood and Tissue kits were purchased from QIAGEN (Hilden, Germany). Lysozyme, gram staining kit (crystal violet, iodine, safranin), ethanol, acetone, Tris-Cl pH 8.0, EDTA, Triton X, and *N*-Hydroxysulfosuccinimide (NHS) were obtained from MilliporeSigma (Burlington, MA). 1-Ethyl-3-(3-dimethylaminopropyl) carbodiimide (EDC), and FluoSpheres™ Carboxylate-Modified Microspheres (100, 200, and 500 nm) were purchased from ThermoFisher Scientific (Walthman, MA). 5 kDa molecular weight (MW) methoxy (MeO)-polyethylene glycol-amine (-NH_2_) was obtained from Creative PEGworks (Chapel Hill, NC).

### Participant enrollment and sample self-collection

All sample self-collection methods were approved by the Johns Hopkins University Institutional Review Board under IRB study IRB00099798, and written informed consent was obtained from each participant. The approved protocol granted access to medical history, which was used to confirm delivery dates, numbers of previous pregnancies, and history of sexually transmitted infections. Participants provided additional information about their medical history and behaviors on a written questionnaire (**Supplementary Figure 1**). Participants (n = 92) were between 18-45 years old and provided a total of n = 432 samples over the course of their pregnancies. In general, samples were collected approximately every 4 weeks during pregnancy. A post-partum sample was collected up to 8 weeks post-delivery. CVM samples were self-collected, as previously described (Boskey et al., 2003; Hoang et al., 2020). Participants were instructed to insert an Instead Softcup^®^ (Evofem) into their vagina and twist while pulling out the cup (< 30 s total) to collect undiluted CVM. They placed the Softcup^®^ into a 50 mL conical tube, which was stored on ice until processing within 12 h of collection. Additionally, participants self-collected a vaginal swab (BD Eswab) sample to be used for sequencing and a urine specimen to confirm pregnancy status with hCG urine test strips.

### Cervicovaginal mucus (CVM) characterization

Conical tubes containing the Softcups were centrifuged at 1000 rcf for 2 min to separate CVM from the Softcup^®^. A Wiretrol^®^ disposable micropipet (Drummond Scientific Co., Broomall, PA) was then used to transfer CVM, which was kept on ice for the duration of the characterization. The pH of the sample was measured using a standard pH microelectrode. A wet mount slide of the sample was imaged to observe bacteria morphology. Gram staining was performed by staining a vaginal smear first with crystal violet, then with iodine. Slides were destained using a mixture of 75% ethanol and 25% acetone, safranin was used as a counterstain (Nugent et al., 1991). Gram stained slides were used for Nugent scoring, which was performed on a color microscope at 100x magnification (Nikon, Tokyo, Japan) (Nugent et al., 1991). The Nugent score relies on gram staining and bacteria morphology to characterize samples as healthy (score 0-3), intermediate (score 4-6), or as having bacterial vaginosis (score 7-10). As described by others, *Lactobacillus*-dominated samples were marked by thick, gram-positive rods (Nugent et al., 1991; Zielinski et al., 2017). The polymicrobial samples (CST IV) showed few rod-shaped bacteria, and many gram-negative cocci. *L. iners* samples showed smaller, gram-variable rods. Lactic acid concentration in the sample was measured using a D/L-lactic acid assay kit, as previously described (Hoang et al., 2020). Briefly, approximately 10 mg of sample was diluted 1:50 with normal saline, and centrifuged at 1000 rcf for 10 min to pellet mucus solids. The supernatant was used in the assay kit according to the manufacturer’s instructions. Cytokine concentrations were measured in one sample from each trimester, from each participant when possible. Samples were stored at -80°C for up to three years, but were freeze-thawed only once. Cytokines were measured using a custom V-PLEX plate, according to the manufacturer’s instructions. IL-1β, IL-6, IL-10, and TNFα were validated to ensure no background interference with CVM. Samples were diluted 1:50 in the supplied sample buffer, and 50 µL of diluted sample was added to appropriate wells. Samples were run in duplicate. Plates were incubated at room temperature on a plate shaker for 2 h. After washing, 25 µL of detection antibody solution was added to each well, and the plate was incubated at room temperature on a plate shaker for 2 h. The plate was washed and 150 µL of read buffer was added to each well. The plate was immediately read using the SECTOR Imager 2400. Cytokine concentration was calculated based on a standard curve run on each plate.

### 16S sequencing and data analysis

DNA extracted from vaginal swab fluid was used to perform 16S rRNA gene sequencing, as previously described (DeLong et al., 2019). Briefly, 150 µL of amies liquid from each swab sample was diluted 5x in lysis buffer and treated with lysozyme for 1.5 h at 37°C prior to processing with a DNeasy^®^ Blood and Tissue, used according to the manufacturer’s instructions. Library preparation and sequencing was performed by the Deep Sequencing and Microarray Core at the Johns Hopkins Medical Institute. The Illumina 16S metagenomic library preparation protocol was used to amplify the V4 region of the 16S gene, and a MiSeq system was used to perform paired-end sequencing of the pooled library. QIIME 2™ was used to generate a table of amplicon sequence variants (ASV) and process 16S sequencing results, as previously described (DeLong et al., 2019). R was used to cluster samples into to community state types (CSTs) using Bray-Curtis distances between all samples and partitioning around the medoids as previously described (DiGiulio et al., 2015). Clustered CSTs were named in accordance with prior convention (Ravel et al., 2011). While we were able to establish delivery dates for all participants, in the case of attrition (n = 11), the last sampled CST was assumed to persist for that participant. In order to adjust for unequal sampling across participants, particle mobility data represents the average particle mobility for all samples within a given CST for a given participant.

### Multiple particle tracking

Multiple particle tracking (MPT) was used to analyze probe particle motion in CVM samples, as previously described (Suh et al., 2005; Lai et al., 2007; Schuster et al., 2015; Hoang et al., 2020). Probe particles used in this study are listed in **Supplementary Table 1**. Replication defective HIV-1 was internally fluorescently labeled with mCherry-Gag, as previously described (Nunn et al., 2015). Fluorescent carboxylate polystyrene beads (conventional particles, CPs) were coated with 5 kDa MW MeO-PEG-NH2 *via* a carboxyl-amine reaction, as previously described (mucus penetrating particles, MPPs) (Nance et al., 2012). Dynamic light scattering was used to measure the size of particles diluted 1:1000 in water (Malvern Zetasizer Nano ZS, 173° scattering angle). Particle ζ-potential (surface charge) was measured at a 1:1000 dilution in 10 mM NaCl. Particles were diluted 10-400-fold depending on particle size in ultrapure water for MPT experiments. For MPT experiments, a 3-dimensional well was made with a small hole puncher (1.5 mm) and two layers of electrical tape on a glass slide. 5 µL of CVM was added to the well, and 0.3 µL of probe particle (HIV, CP, or MPP) was added to the CVM sample. A glass coverslip and superglue were used to seal the well. Particle motion was recorded in a 20 s video, taken at room temperature using an EM-CCD camera (Evolve 512; Photometrics) mounted on a Zeiss Axio Observer inverted epifluorescence microscope. Videos were taken using the 100x oil-immersion objective, had an image resolution of 25 nm/px, and a frame rate of 15 Hz. For each CVM sample, a minimum of 5 videos were taken for each particle type. Particle motion in CVM samples was quantified using a MATLAB image processing script, as previously described (Suh et al., 2005; Lai et al., 2007; Schuster et al., 2015; Hoang et al., 2020). The MATLAB output included a mean squared displacement (MSD) for each individual particle, which was used to generate a histogram of all particle MSDs at a time scale of τ = 1 sec with the normalmixEM tool (R mixtools version 1.2.0). Using this histogram, R was used to find representative Gaussian distributions, indicative of a fraction of trapped particles (represented by lower MSDs) and a fraction of mobile particles (higher MSD). In cases where the Gaussian distribution revealed only one peak, a log(MSD) of -1.3 µm^2^ was used as a cutoff to distinguish between trapped and mobile particles. The cutoff value was selected based on providing a clear separation between the two Gaussian curves for all particle types, and was in a similar range of values that separated the curves describing a slower and a faster moving particle population for a variety of nanoparticle types in human mucus secretions (Ensign et al., 2012; Schuster et al., 2014; Schuster et al., 2015). Average MSD at τ = 1 sec data were used to calculate the pore size of each CVM sample, as previously described (Lai et al., 2010). Here, we utilized data from 100, 200, and 500 nm MPPs to calculate an average pore size for each sample.

### Statistical analysis

Samples were excluded if they contained blood, or if a participant marked that they had engaged in unprotected vaginal intercourse in the three days prior to sample collection. GraphPad Prism 8 (San Diego, CA) was used for statistical analyses. Simple linear regressions were done on scatter plots to determine if the slope was significantly non-zero. For these analyses, both R^2^ and *p*-value are reported. Student’s t-tests (comparison of 2 groups) and one-way ANOVA corrected using Tukey’s multiple comparisons test (for comparisons of 3 or more groups) were used to compare particle mobility and cytokine concentrations. Unless otherwise noted, significance is shown as *p* < 0.05. Relative risk, odds ratio, and 95% confidence intervals were calculated in GraphPad Prism 8. Data are presented as mean ± SD.

## Results

### Participant demographics differ between term delivery and preterm delivery cohorts

Participant demographics based on self-reported questionnaires and medical records are tabulated in **Table 1**. The median age of participants was 29, with a range from 18-41. Twenty-four (26%) participants identified as Hispanic or Latino, 40 (43%) were white, 25 (27%) were Black or African-American, 4 (4%) were Asian, and 23 (25%) self-identified as ‘other’. Twenty-four (26%) of participants self-reported a diagnostic history of female reproductive tract conditions. Seventy-one (77%) participants self-reported a previous pregnancy, with 7 (8%) reporting a previous PTB, and 26 (28%) reporting a previous miscarriage. Based on patient electronic medical records (EMR), 26 (28%) participants had previously experienced homelessness, and 35 (38%) had experienced intimate partner violence. During their current pregnancies, 16 (17%) participants reported BV symptoms, while 9 of the 16 participants who reported BV symptoms reported having received treatment for BV. Fourty-eight (52%) participants were considered high-risk in the EMR system. Of high-risk participants, 85% (n = 41/48) were part of an addiction counseling program.

**Table 1:** Demographics and characteristics for participants enrolled in the longitudinal analysis of mucosal barrier properties and vaginal microbiome during pregnancy.

Pregnancy demographics	Total Cohort (n=92)	Term (n=82)	Preterm (n=10)	Relative Risk	Odds Ratio	95% CI	p Value
	Median (range)						
Age	28.8 (18-41)	28.9 (18-41)	28.3 (22-35)
BMI	29.9 (18-64)	30 (18-64)	28.8 (22-38)
Weeks at delivery	38.7 (27-43)	39.3 (37-43)	34.4 (27-37)
Ethnicity	Number (%)		
Hispanic or Latina	24 (26)	22 (27)	2 (20)	0.7083	0.6818	0.14 to 3.3	>0.999
Race	Number (%)						
White	40 (43)	39 (48)	1 (10)	0.144	0.123	0.011-0.84	**0.039**
Black or African American	25 (27)	18 (22)	7 (70)	6.25	8.296	0.032-0.53	**0.004**
Asian	4 (4)	4 (5)	0 (0)	0	0	0.0-9.55	>0.999
Other	23 (25)	21 (26)	2 (20)	0.781	0.726	0.146-3.5	>0.999
History of conditions	Number (%)						
None	68 (74)	63 (77)	5 (50)	0.353	0.302	0.085-1.09	0.12
Yes, specified (see below)	24 (26)	19 (23)	5 (50)	2.833	3.32	0.919-11.77	0.12
HIV	2 (2)	1 (1)	1 (10)	5	9	0.429-172.1	0.21
Herpes	6 (7)	5 (6)	1 (10)	1.59	1.71	0.132-11.75	0.51
Gonorrhea	3 (3)	1 (1)	2 (20)	7.42	20.25	2.03-294.8	**0.03**
Chlamydia	10 (11)	7 (9)	3 (30)	3.51	4.59	1.074-19.81	0.075
Bacterial vaginosis (BV)	13 (14)	10 (12)	3 (30)	2.6	3.09	0.758-11.82	0.147
Syphilis	1 (1)	1 (1)	0 (0)	0	0	0.0-73.80	>0.999
Trichomoniasis	9 (10)	8 (10)	1 (10)	1.03	1.03	0.0841-7.19	>0.999
History of experiences	Number (%)						
Previous pregnancy	71 (77)	68 (83)	3 (30)	0.127	0.088	0.038-0.419	**0.0010**
Previous PTB	7 (8)	5 (6)	2 (20)	3.04	3.85	0.67-20.98	0.166
Previous miscarriage	26 (28)	23 (28)	3 (30)	1.09	1.10	0.29-4.63	>0.999
Previous BV	18 (20)	16 (20)	2 (20)	2.60	3.09	0.76-11.82	0.147
Previous short cervix	1 (1)	1 (1)	0 (0)	0	0	0.0-73.8	>0.999
Previous low birth weight	4 (4)	4 (5)	0 (0)	0	0	0.0-9.55	>0.999
Previously homeless	26 (28)	22 (27)	4 (40)	1.69	1.82	0.53-6.37	0.460
Experienced intimate partner violence	35 (38)	30 (37)	5 (50)	1.62	1.73	0.50-5.94	0.496
Current pregnancy experiences	Number (%)						
BV Symptoms	16 (17)	15 (18)	1 (10)	0.5278	0.4963	0.04263-3.762	>0.9999
BV Treatment	9 (10)	9 (11)	0 (0)	0	0	0.0-3.669	0.5897
High risk	48 (52)	43 (52)	5 (50)	0.9167	0.907	0.2661-3.096	>0.9999
Addiction counseling	41 (45)	38 (46)	3 (30)	0.5331	0.4962	0.1332-2.026	0.5029
Smoking	40 (43)	38 (46)	2 (20)	0.325	0.2895	0.05965-1.339	0.1774
Male baby	47 (51)	40 (49)	7 (70)	2.234	2.45	0.6000-9.109	0.3167

Significance is indicated by a bolded p Value.

**Figure 1:**
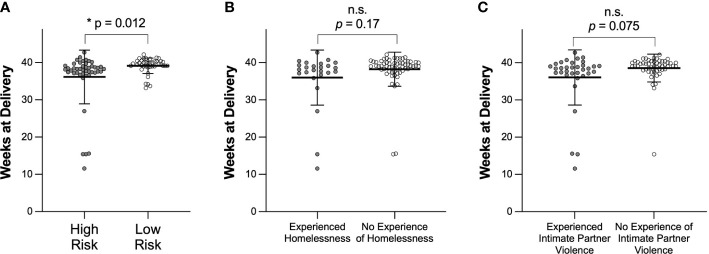
Comparison of weeks of pregnancy at time of delivery based on **(A)** clinical designation of high or low risk, **(B)** experience of homelessness, or **(C)** experience of intimate partner violence. Data are shown as mean ± SD. Significance was determined using a t test with Welch’s correction. n.s. indicates not significant.

**Table 1** also shows demographics for participants who delivered prematurely. We observed 10.8% (10/92) of participants delivering prematurely in this study, consistent with the US average of ~10% (Cunningham et al., 2018; Mod, 2019). The median age of participants who delivered prematurely was 28, with a range of 22-35. The median BMI was 28.8, and the median delivery time was 34.4 weeks. In contrast to the total cohort, 70% of participants who delivered prematurely were Black or African American (*p* = 0.004). While only 3/10 participants who delivered prematurely had experienced a prior pregnancy, 2/3 of these participants had experienced a prior premature delivery. **Figure 1** compares weeks at delivery for participants determined clinically to be high- or low-risk (**Figure 1A**), participants who had or had not experienced homelessness (**Figure 1B**), and participants who had or had not experienced intimate partner violence (**Figure 1C**). Participants who were clinically high-risk had a significantly earlier delivery date than participants who were low-risk (*p* = 0.012). There were no significant differences in participants who had or had not experienced homelessness or intimate partner violence, although both groups of participants who had experienced homelessness and those who had experienced intimate partner violence trended towards shorter median weeks of pregnancy at delivery than participants who had not.

Participants in this study provided a total of 432 samples. Based on exclusion criteria, 377 samples were used in analyses for mucus changes during pregnancy, where 17 participants provided 1 sample each, 15 participants provided 2 samples each, 12 participants provided 3 samples, 8 participants provided 4 samples, 13 participants provided 5 samples, 11 participants provided 6 samples, 6 participants provided 7 samples, 4 participants provided 8 samples, 4 participants provided 9 samples, 1 participant provided 10 samples, and 1 participant provided 11 samples.

### Weeks of pregnancy does not predict cervicovaginal mucus (CVM) barrier properties

**Figure 2:**
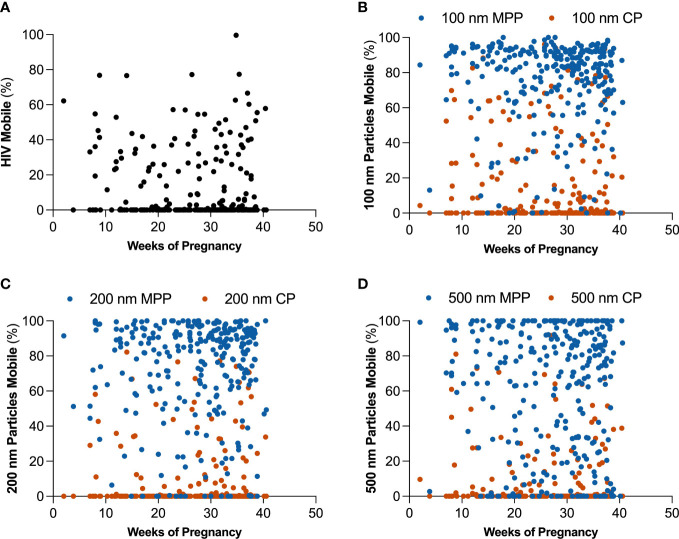
Changes in estimated percentage of mobile particles at τ = 1 s over the course of pregnancy for **(A)** HIV virion, **(B)** 100 nm mucus penetrating particles (MPP) in blue and 100 nm conventional particles (CP) in orange, **(C)** 200 nm MPP in blue and 200 nm CP in orange, and **(D)** 500 nm MPP in blue and 500 nm CP in orange.

We sought to investigate how pregnancy may impact mucus barrier properties. Using MPT, we quantified probe particle motion in CVM samples self-collected by participants approximately every 4 weeks from study enrollment. We first compared particle mobility in all CVM samples collected in this study. **Supplementary Figure 2** shows differences in HIV, as well as CP and MPP mobility for 100, 200, and 500 nm particles. HIV showed 15.7 ± 21.35% mobility across all samples. CPs are both adhesively and sterically hindered by interactions with mucus, and MPPs are trapped only *via* steric interactions. Consistent with what previous observations in CVM, CP were less mobile than MPPs across 100 (18% v 78%), 200 (15% v 77%), and 500 nm (8% v 65%) particle sizes. Additionally, we observed a significant decrease in 500 nm CP and MPP mobility as compared to 100 and 200 nm CP and MPP, respectively. **Figure 2** demonstrates that there were no significant trends in overall particle mobility over the course of pregnancy. Further, we demonstrate in **Supplementary Figure 3** and **Supplementary Table 2** that using MPP mobility as an indicator of the surrounding mesh structure, the average pore size in the CVM was 309 nm, consistent with our previous findings in CVM from non-pregnant women (Lai et al., 2010).

### Pregnancy outcomes are linked to composition of the vaginal microbiome

**Figure 3:**
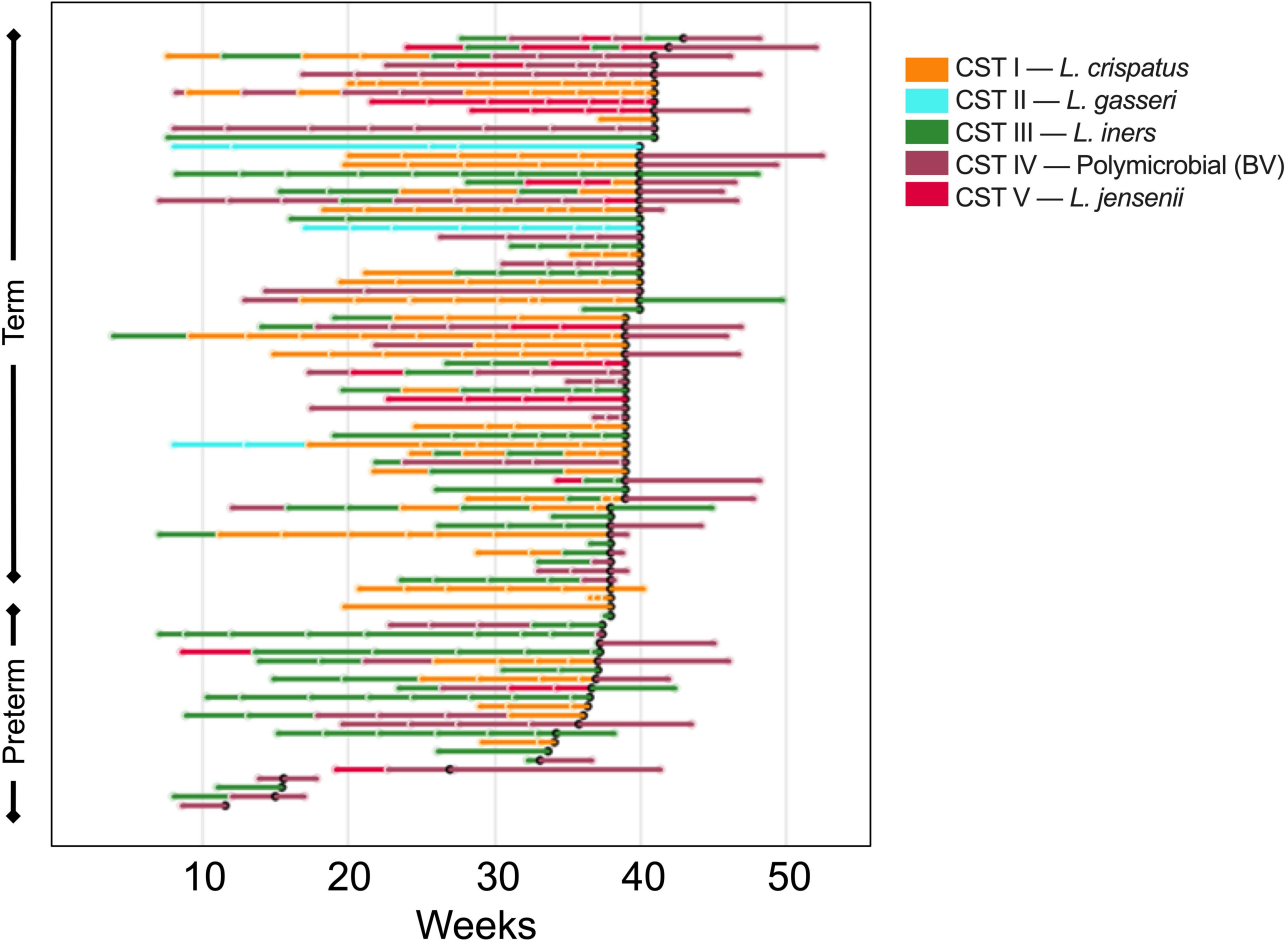
Changes in vaginal microbiome CST for each participant in the study over the course pregnancy. The lines are colored to correspond to the CST of the prior sampling, and each break in the line indicates a new sample. The bar color indicates the CST as shown in the key, where orange represents CST I, blue CST II, green CST III, purple CST IV, and pink CST V. The black dot represents time of delivery, and the lines following the black dot represent the postpartum sample.

We next determined the composition of the vaginal microbiome for each sample collected in this study. We utilized 16S rRNA gene sequencing (75,363 ± 1,729 reads per sample) to assign each sample into one of five previously defined community state types (CSTs): CST I was marked by a dominance of *L. crispatus*; CST II by a dominance of *L. gasseri*; CST III by *L. iners*; CST IV included samples which were polymicrobial; and CST V by *L. jensenii* (Ravel et al., 2011). A heatmap depicting the relative abundance of bacteria species measured in each sample can be found in **Supplementary Figure 4**. Of the 432 samples collected in this study, 29% were characterized as CST I (n = 125); 4% CST II (n = 16); 32% CST III (n = 138); 28% CST IV (n = 122); 7% CST V (n = 30). One sample was unable to be sequenced due to lack of sample. **Figure 3** depicts samples from each individual participant, grouped by whether the participant delivered at full term or delivered prematurely, and shows the breakdown of CSTs for samples collected over time. Consistent with what was previously described, many participants in our study experienced shifts in CST over time, with shifts from CST III to CST I, and CST III to CST IV being the most common (**Figure 3**).

**Figure 4:**
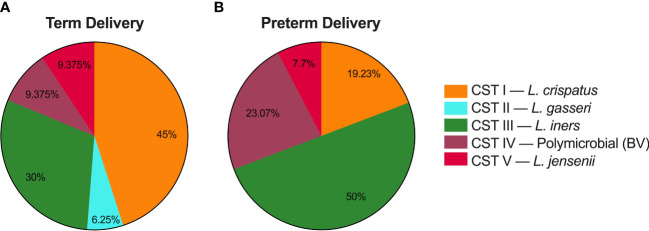
Delivery times based on CVM microbial composition for samples collected as part of this study. **(A)** Percentage of samples from participants who delivered at term that were characterized as community state type (CST) I, CST II, CST III, CST IV, and CST V. **(B)** Percentage of samples from participants who delivered prematurely that were characterized as each CST.

After assigning each sample to a CST, we analyzed PTB risk based on composition of the vaginal microbiome across pregnancy, as previous work demonstrated that CST IV was associated with an increased risk of PTB (Romero et al., 2014a; DiGiulio et al., 2015; Koullali et al., 2016; Callahan et al., 2017; Kindinger et al., 2017; Amabebe and Anumba, 2018; Elovitz et al., 2019). **Figure 4** shows the percentage of all samples collected that were classified in each CST for participants who delivered at term (**Figure 4A**), or preterm (**Figure 4B**). In participants who delivered at term, 45% of all samples collected were CST I, 6.2% CST II, 30% CST III, 9.4% CST IV, and 9.4% CST V. In participants who delivered prematurely, 19.2% of samples were CST I, 0% CST II, 50% CST III, 23.1% CST IV, and 7.7% CST V.

### The composition of the vaginal microbiome is the dominant factor associated with changes to the physical properties of CVM during pregnancy

We previously observed that the physical properties of CVM from non-pregnant participants are affected by the composition of the vaginal microbiome (Zierden et al., 2020). **Supplementary Figure 5** shows the pH, Nugent score, total lactic acid, and total D-lactic acid for samples collected from our pregnant cohort classified in each CST. These data are consistent with what we have previously observed in samples from non-pregnant participants, where CST IV was characterized by an increased pH, increased Nugent score, and decreased lactic acid content. Similarly, CST III (*L. iners* dominated) showed decreased D-lactic acid, consistent with previous reports that *L. iners* produces only L-isoform of lactic acid. We further demonstrate, in **Supplementary Figure 6**, that the pH of the sample was significantly correlated with Nugent score, total lactic acid, and D-lactic acid.

**Figure 5:**
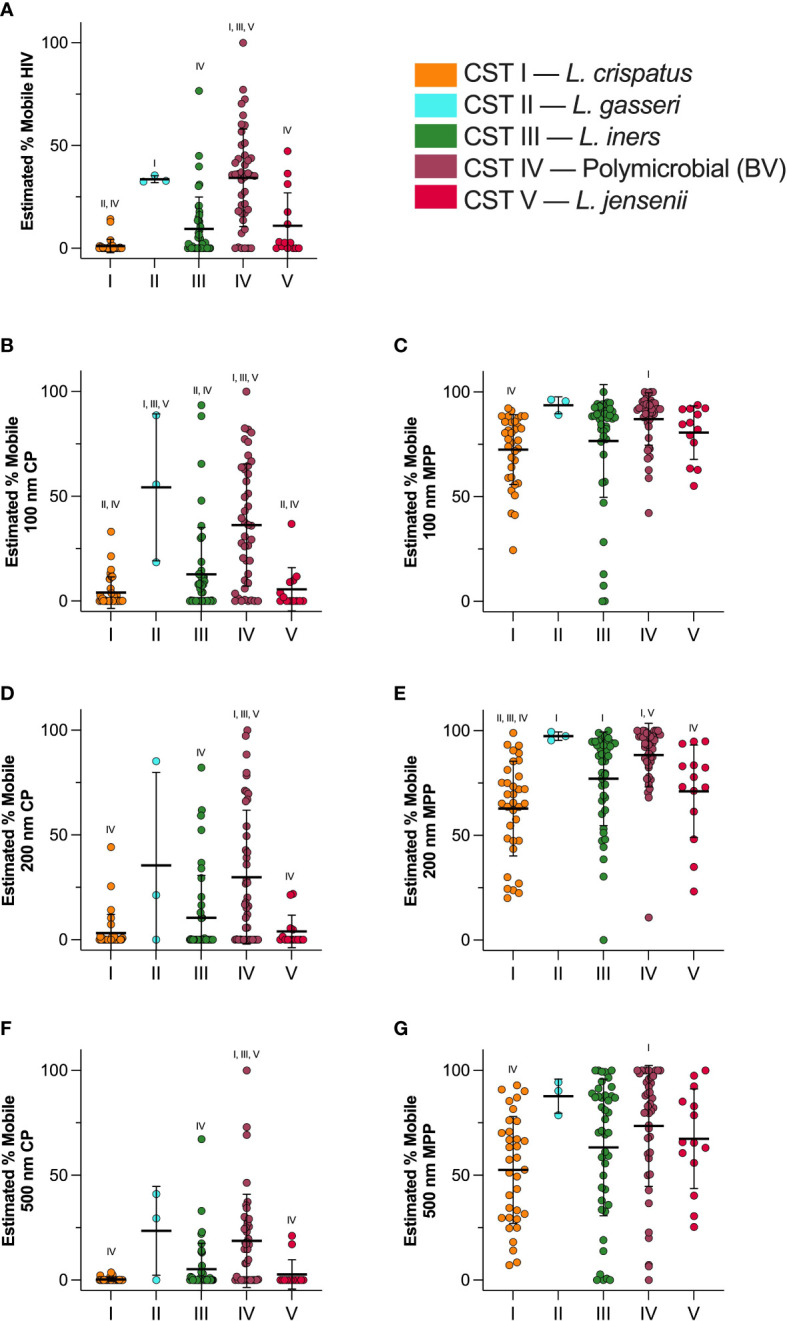
Estimated percentage of mobile particles at τ = 1 s in each CST. **(A)** HIV virion mobility, **(B)** 100 nm CP mobility, **(C)** 100 nm MPP mobility, **(D)** 200 nm CP mobility, **(E)** 200 nm MPP mobility, **(F)** 500 nm CP mobility, **(G)** 500 nm MPP mobility. Data are shown as mean ± SD. Significant differences are represented by CST groups listed above each dataset (*p* < 0.05). Significance was determined using ANOVA with Tukey’s multiple comparison test.

**Figure 5** shows particle mobility in samples classified in each CST. These data are represented as log_10_(MSD) at τ = 1 s in **Supplementary Figure 7**. Across all particle types, CST I showed most trapping, and CST II showed least trapping. Consistently, CST IV showed reduced barrier properties as compared to CST I. CST I showed almost complete trapping of HIV (1.1 ± 6.6% mobile), and the average percentage of mobile particles increased from CST III < CST V < CST II < CST IV up to 34.3 ± 23.7% (**Figure 5A**). The percentage of mobile HIV particles in CST I samples was significantly reduced compared to CST II (*p* < 0.014) or CST IV (*p* < 0.0001) samples, and the percentage of mobile HIV particles was significantly increased in CST IV samples compared to CST III (*p* < 0.0001), and CST V (*p* = 0.0001) samples. **Figure 5B** shows mobility of 100 nm CP. CST I showed the most trapping at 4.0 ± 7.5% mobility, whereas CST II showed the least trapping at 54.3 ± 35.1% mobility (**Figure 5B**). 100 nm CP were significantly less mobile in CST I samples than in CST II (*p* = 0.0018) or CST IV (*p* < 0.0001), whereas CST IV samples showed decreased hindrance of 100 nm CP as compared to CST III (*p* < 0.0001), and CST V (*p* = 0.0002). The estimated percentage of 100 nm MPP in each CST is shown in **Figure 5C**. CST I showed the most trapping at 72.5 ± 16.7% mobility, whereas CST II showed 93.7 ± 4.0% mobility. CST IV showed 87.1 ± 12.5% mobility. 100 nm CP were significantly less mobile in CST I samples than in CST IV (*p* = 0.012). **Figure 5D** shows mobility of 200 nm CP. CST I showed the least mobility at 3.2 ± 8.9%, whereas CST II showed the most mobility at 35.5 ± 44.4%. 100 nm CP were significantly less mobile in CST I samples than in CST IV (*p* < 0.0001), and CST IV samples showed decreased hindrance of 200 nm CP as compared to CST III (*p* = 0.0012), and CST V (*p* = 0.0026). The 200 nm MPP mobility for each CST is shown in **Figure 5E**. CST I showed 62.8 ± 22.6% mobility, followed by CST V, CST III, CST IV, and finally CST II with 97.4 ± 2.0% mobility. 200 nm MPP were significantly more mobile in CST IV than in CST I (*p* < 0.0001) and CST V (*p* = 0.049). **Figure 5F** shows mobility of 500 nm CP where CST I showed 0.31 ± 0.86% mobility. CST I < CST V < CST III < CST IV < CST II at 23.5 ± 21.2% mobility. CST IV samples showed decreased hindrance of 500 nm CP as compared to CST I (*p* < 0.0001), CST III (*p* = 0.0004), and CST V (*p* = 0.0052). Finally, 500 nm MPP estimated mobility is shown in **Figure 5G**. CST I showed 52.6 ± 25.5% mobility. Particle mobility was the highest in CST II at 87.7 ± 8.1%. 100 nm CP were significantly less mobile in CST I samples than in CST IV samples (*p* < 0.0001). As expected, particle mobility is significantly positively correlated with sample pH (**Supplementary Figure 8**). Furthermore, we observed a significant difference in average pore size between CST I and CST IV samples (**Supplementary Figure 9**). Consistent with **Figure 2**, there were no significant differences in particle mobility over the course of pregnancy when samples were separated by CST (**Supplementary Figure 10**).

### Cytokine concentrations in CVM did not correlate with weeks of pregnancy or CST

**Figure 6:**
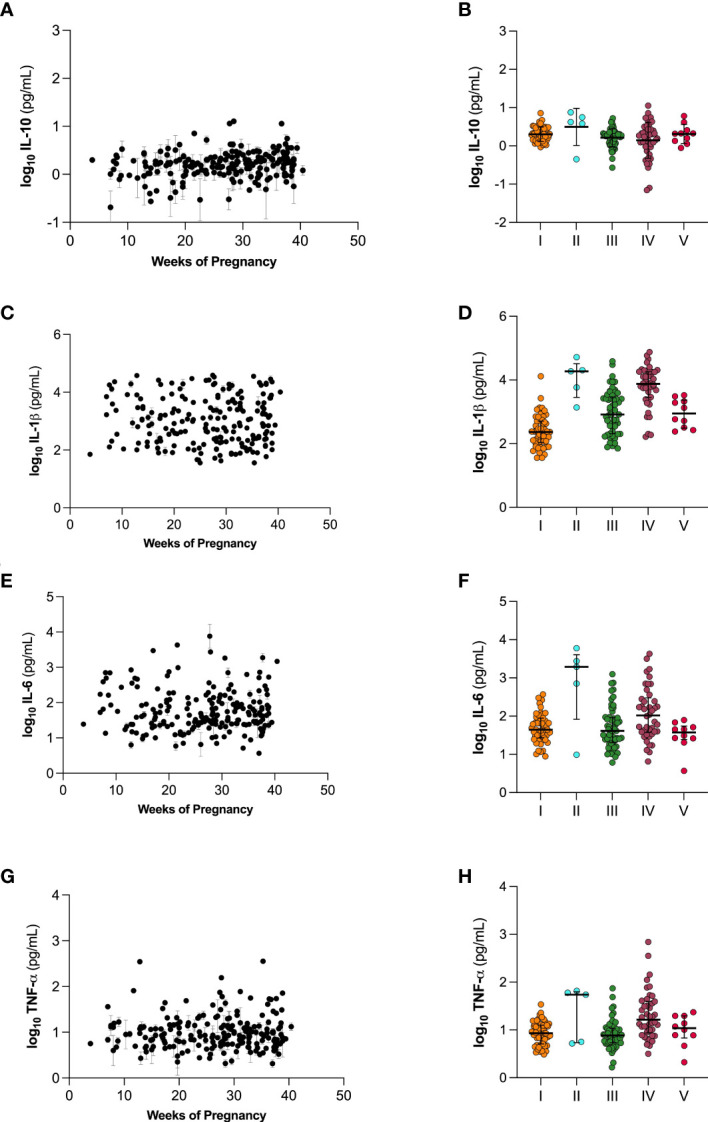
Cytokine levels over the course of gestation **(A, C, E, G)** and separated by vaginal microbiome CST **(B, D, F, H)**. **(A, B)** IL-10, **(C, D)** IL-1β, **(E, F)** IL-6, and **(G, H)** TNF-α. Individual datapoints in **(A, C, E, G)** are shown as mean ± SEM based on technical replicates. Data in **(B, D, F, H)** are shown as mean ± SD.

We next investigated cytokine concentration in the five CSTs across pregnancy. **Figure 6** shows changes in IL-10 (**Figure 6A**), IL-1β (**Figure 6C**), IL-6 (**Figure 6E**), and TNF-α (**Figure 6G**) over the course of pregnancy. We observed no significant trends in the changes of cytokine levels over the course of pregnancy. **Figure 6** also shows differences in cytokine levels between the five CSTs. We observed no significant differences in IL-10, IL-1β, IL-6, or TNF-α in CVM samples across the CSTs.

## Discussion

The human body is equipped with a mucus barrier that protects exposed epithelial tissues (Cone, 2009). Mucus is comprised of crosslinked glycoproteins that form a net-like structure capable of trapping and eliminating foreign pathogens from the body (Cone, 2009). This mucus mesh has small pores that sterically hinder bacteria and large pathogens from reaching the underlying epithelium (Lai et al., 2010). Additionally, mucin proteins are comprised of hydrophobic and negatively charged regions that serve to adhesively trap particulates (Cone, 2009). Mucus is continuously secreted, facilitating the clearance of foreign entities that may have been trapped either sterically or adhesively by the mucus mesh (Cone, 2009). In the female reproductive tract, CVM protects women from sexually transmitted infections, such as herpes simplex virus (HSV) and HIV-1, and from bacteria that can lead to urinary tract infections (UTIs), pelvic inflammatory disease (PID), and intrauterine infection (Cone, 2009; Lai et al., 2009a; Nunn et al., 2015; Hoang et al., 2020). In the context of pregnancy, CVM further serves to protect the developing fetus from infection that may lead to inflammation, resulting in adverse fetal outcomes and PTB (Howe et al., 1999; Cone, 2009; Koullali et al., 2016; Amabebe and Anumba, 2018). Despite these benefits, the vaginal mucus barrier also serves as a challenge to effective vaginal drug administration. We have demonstrated that vaginal drug delivery is optimal for targeting the female reproductive tract and preventing PTB in preclinical mouse models (Hoang et al., 2019; Zierden et al., 2021a). Here, we characterized CVM barrier properties and the vaginal microbiome over the course of pregnancy in order to define design criteria for vaginally administered therapeutics for obstetric indications. Importantly, the participants included in this study represent a wide range of race and ethnicities, as well as socioeconomic backgrounds.

Vaginal drug delivery increases local drug levels, while minimizing unwanted, off-target side effects (Ensign et al., 2014). Additionally, the uterine first pass effect facilitates recirculation of drug around the uterus, before reaching systemic circulation (De Ziegler et al., 1997). However, as an adhesive net designed to trap foreign pathogens, CVM also serves as a barrier to effective vaginal drug delivery (Cone, 2009). While many studies explore the relationship between the vaginal microbiome and pregnancy outcomes for both mother and neonate, few studies describe changes to mucus structure over the course of pregnancy. During the normal 28-day menstrual cycle, CVM properties change based on fluctuating levels of progesterone and estrogen (Moreno-Escallon et al., 1982). As estrogen increases, and the body prepares for ovulation, the water content of cervical mucus increases and the average pore size increases, to facilitate sperm transport through mucus (Mihm et al., 2011). As progesterone increases in the luteal phase, mucus thickens, and the effective pore size decreases (Moreno-Escallon et al., 1982; Mihm et al., 2011). During pregnancy, endogenous progesterone levels increase until the point of delivery. Characterization of cervical mucus collected from the external cervical os, using shear rheology and particle permeability experiments revealed that samples collected from high-risk patients showed increased spinnbarkeit and weakened crosslinking (Critchfield et al., 2013). Furthermore cervical mucus collected from participants considered high-risk was more permeable to 200 nm particles (Critchfield et al., 2013). These data are important for potentially identifying patients at risk for PTB, but do not consider vaginal microbiota, which we show plays a role in mucus barrier properties. Here, we used multiple particle tracking (MPT) with both CPs and MPPs in fresh, undiluted CVM samples, to probe adhesive properties and pore size of CVM over the course of pregnancy and in the context of the vaginal microbiome. MPPs are engineered to be coated with a high density of low-molecular weight polyethylene glycol (Nance et al., 2012). This coating results in a net neutral surface charge, and shields the hydrophobic polystyrene nanoparticle from adhesive interactions with the mucin proteins, so while conventional particles (CPs) are trapped adhesively by mucins, tracking MPPs allows us to measure steric interactions between the mucus mesh and particles of various sizes (Lai et al., 2009b; Lai et al., 2010). We previously used this technique to demonstrate that the average pore size of CVM self-collected by reproductive age, non-pregnant participants was ~340 ± 70 nm, much larger than the previous estimate of ~100 nm average size (Lai et al., 2010).

We previously demonstrated that utilizing MPP technology, we can engineer drug-loaded nanoparticles for effective vaginal drug delivery that provide improved drug delivery to the female reproductive tract compared to clinically used formulations (Ensign et al., 2013; DeLong et al., 2019). In a second study, we demonstrated that effective drug delivery can reveal mechanisms of inflammation-induced PTB, and help to identify therapeutics capable of preventing inflammation-induced PTB (Zierden et al., 2020; Zierden et al., 2021a). In a murine model of inflammation-induced PTB, we demonstrated that a novel combination of vaginally delivered drug-loaded MPPs was able to prevent PTB, whereas systemic administration of the same drug combination was ineffective (Zierden et al., 2021a). Here, using probe nanoparticles, and fluorescently labeled virions, we observed how particle mobility was affected by the heterogenous network of mucin proteins (Lai et al., 2009b). Consistent with studies using samples from non-pregnant participants, we observed that the mobility of 500 nm MPP was significantly more hindered than either 100 nm MPP or 200 nm MPP. However, we observed no significant impact of the longitudinal time throughout pregnancy on the barrier properties of CVM. Rather, we observed that the composition of the vaginal microbiome was more impactful. We used 16S rRNA gene sequencing to assign each sample to a community state type (CST) (DiGiulio et al., 2015; Gosmann et al., 2017; DeLong et al., 2019). As we previously observed in a non-pregnant cohort, particles and virions were more trapped in samples with a dominance of *L. crispatus* (CST I), as compared to polymicrobial samples (CST IV). Using MPP mobility to calculate the distribution of pore sizes in the surrounding environment, we observed that the average pore size in CVM from pregnant participants did not change over the course of pregnancy, and that the pore size was similar to that observed in prior studies characterizing CVM from non-pregnant participants (Lai et al., 2010). However, CVM from women with microbiota classified as CST I showed a smaller average pore size than that of CST IV, consistent with particle mobility data.

We have previously described that *ex vivo* particle mobility was predictive of vaginal particle distribution *in vivo* (Ensign et al., 2012). Here, our data demonstrate that for a vaginally delivered nanoformulation to be most effective, taking into consideration the most stringent case (CST I, first trimester), the particles should be mucus penetrating and smaller than 500 nm in size (Ensign et al., 2012; Ensign et al., 2013). Furthermore, as microbiota have the greatest effect on CVM barrier function, formulations should not perturb the vaginal environment and the microbiota. This includes having a low pH (~4.0), and not being hypertonic, which would cause irritation and inflammation in the vaginal compartment (Ensign et al., 2013; Zierden et al., 2021a; Zierden et al., 2021b). Vaginal formulations incorporating these criteria are likely to provide more efficient drug delivery to treat indications during pregnancy, including the prevention of PTB.

Many studies have investigated the human vaginal microbiome during pregnancy, and its implications in PTB (Romero et al., 2014a; Romero et al., 2014b; Elovitz et al., 2019; Gerson et al., 2019; Serrano et al., 2019). Generally, *Lactobacillus* spp. are regarded as beneficial, and are associated with term deliveries. In contrast, patients with CST IV (polymicrobial, BV) vaginal microbiota are more likely to experience PTB than patients with vaginal microbiota dominated by *L. crispatus* (DiGiulio et al., 2015). It is hypothesized that BV leads to decreased barrier function of CVM, allowing the ascension of bacteria or other inflammatory stimuli from the vaginal environment into the uterine compartment. Clinically, patients with BV often report an excess of thin, watery discharge. Indeed, others measured a decrease in mucus viscosity associated with BV using macrorheology (Chappell et al., 2014). Here, we demonstrated that, regardless of timing of pregnancy, CST IV samples showed decreased barrier properties as compared to CST I samples. We previously observed that CST III samples show decreased barrier properties (Zierden et al., 2020). Clinically, women with CST III vaginal microbiota are also at an increased risk for PTB (Kindinger et al., 2017). While CST III did not show significant differences in HIV and CP mobility as compared to CST I, we observed a significant increase in mobility of 200 nm MPP and 500 nm MPP. Increased mobility of 200 nm and 500 nm MPPs in CST III is indicative of larger pore sizes, which are ultimately less protective than smaller pores which have the ability to immobilize a wider range of pathogens, fitting with our previous findings regarding *L. iners*-dominated CVM (Hoang et al., 2020).

One potential mechanism by which CVM barrier properties are diminished is *via* degradation of mucins by BV-associated bacteria. In mice, it has been shown that *G. vaginalis*, a BV-associated species, is associated with an increase in sialidases and other enzymes capable of breaking down the CVM barrier (Howe et al., 1999; Wiggins et al., 2001; Cauci et al., 2008; Gilbert et al., 2013; Lewis et al., 2013). In mice, vaginal inoculation with *G. vaginalis* led to increased levels of IL-6, IL-10, IL-1β, and TNF-α (Anton et al., 2018; Sierra et al., 2018). *Mobiluncus mulieris*, another species highly associated with BV, has been shown to upregulate IL-6 and IL-8 in culture (Elovitz et al., 2019; Dude et al., 2020). While we observed no significant differences in the cytokine concentration of CVM samples in this cohort, we hypothesize this may be attributed to differences in the method of sample collection. Further, this may be related to molecular characterization of CST IV, rather than characterization *via* Amsel’s criterion or clinical diagnosis with symptomatic BV.

While our study established a connection between the vaginal microbiota and altered mucus barrier properties during pregnancy, incomplete longitudinal data and participant attrition limited the ability to find longitudinal associations. The diversity of the participant demographics was a strength of the study, but the sample size was too small to make conclusions about how participant demographics may affect PTB rates. Previous work associated race and ethnicity with risk for PTB, where Black and African American patients, as well as Hispanic patients, are at a higher risk for adverse obstetric outcomes (Alcendor, 2016; Callahan et al., 2017). Similarly, Black and African American women have an increased for being diagnosed with BV (Alcendor, 2016). Additionally, there is evidence that socioeconomic status, intimate partner violence, and stress affect the composition of the vaginal microbiome and pregnancy outcomes, further compounding observed trends from a human dataset (Turpin et al., 2021). Several larger studies integrated data from thousands of samples to identify important associations between vaginal microbiota, mucosal cytokines, demographics, and timing of parturition (Fettweis et al., 2019; Serrano et al., 2019).

The work here quantifies a mucus pore size in relation to time of pregnancy, as well as the vaginal microbiome. Our data demonstrate that the vaginal microbiome was the best predictor of CVM barrier properties. These results support what has been shown in recent studies, that not having a *L. crispatus* dominated vaginal microbiome is a risk factor for PTB (Kindinger et al., 2017). PTB is a global issue, impacting 15 million pregnancies each year (Cunningham et al., 2018). Infants born prematurely experience a wide range of health burdens, including necrotizing enterocolitis, retinopathy of prematurity, cerebral palsy, hearing impairments, among others. Currently, a sonographic short cervix, and a history of premature delivery, are the only two risk factors used to identify a woman at risk for PTB. It is hypothesized that the bacteria from the polymicrobial environment can ascend into the uterine environment, leading to inflammation and prematurely initiating biological mechanisms of parturition. Here, we use multiple particle tracking (MPT) to demonstrate that polymicrobial CVM samples from pregnant participants have significantly diminished cervicovaginal mucus (CVM) barrier properties, as compared to participants with *L. crispatus* dominated microenvironments. Our findings contribute to the potential for analyzing CVM properties to identify patients at risk for PTB, and further define design criterion for engineering therapeutics for vaginal delivery during pregnancy.

## Data availability statement

The data presented in the study are deposited in the Sequence Read Archive (SRA) repository, accession number PRJNA942697, found at: https://www.ncbi.nlm.nih.gov/bioproject/PRJNA942697. All other relevant data are included within the paper and supplemental information.

## Ethics statement

The studies involving human participants were reviewed and approved by Johns Hopkins University Institutional Review Board. The patients/participants provided their written informed consent to participate in this study.

## Author contributions

HZ, KD, and LE conceived and designed the study. HZ, FZ, VL, SB, NH, JO, and TH performed experiments. KD conducted statistical analyses. SL provided HIV-1 fluorescently labeled with mCherry-Gag. AB, JO, and HZ enrolled participants. HZ, KD, JH, and LE interpreted results. All authors contributed to the article and approved the submitted version.
