# Supplementary material for: Cervicovaginal mucus barrier properties during pregnancy are impacted by the vaginal microbiome
Source: Front Cell Infect Microbiol. 2023 Mar 31;13:1015625. doi: 10.3389/fcimb.2023.1015625 (PMC10103693; doi:10.3389/fcimb.2023.1015625)
Supplement: Supplementary file 1 [file DataSheet_1.pdf]

Supplementary Material

Table S1: Probe particle size (nm) and **ζ**-potential (mV) used in multiple particle tracking experiments.

**Particle**	**Size** (nm)	**ζ-potential** (mV)
100 nm PS (100 CP)	116 ± 1	-53.3 ± 1.65
100 nm PS-PEG (100 MPP)	134 ± 4	-0.576 ± 0.569
200 nm PS (200 CP)	205 ± 3	-40.0 ± 1.70
200 nm PS-PEG (200 MPP)	246 ± 7	-2.80 ± 0.112
500 nm PS (500 CP)	523 ± 34	-37.6 ± 4.35
500 nm PS-PEG (500 MPP)	566 ± 37	-7.79 ± 0.174

QUESTIONNAIRE (pregnant participants)
(IRB Application #IRB00099798)

Date ____________________________**Participant #** ___________________________

Personal Data[1]

1. Please provide your age: _____________
2. Please indicate your ethnicity (circle one):
   a. Hispanic or Latino   b. Not Hispanic or Latino
3. Please indicate your race (circle one):
   a. American Indian or Alaska Native   b. Asian
   c. Native Hawaiian or Other Pacific Islander   d. Black or African American
   e. White   f. Other

Questionnaire (Please circle "Yes" or "No" and fill in blanks where applicable)[2]

1. How many weeks pregnant are you? ____________ weeks
2. Is this your first pregnancy? Yes No
3. Have you ever experienced (check all that apply)?
   _____miscarriage _____ sonographic short cervix _____ preterm birth
   _____bacterial vaginosis (**BV**) _____infection of amniotic fluid _____low birth weight
4. If you have delivered preterm before, how many weeks were you at delivery? _________ weeks
5. Have you had symptoms of BV during your pregnancy (runny or unusual discharge, unusual odor sometimes described as "fishy")? Yes No
6. Have you been treated and/or diagnosed with BV during your pregnancy? Yes No
7. Are you expecting twins, triplets, or other multiples? Yes No
8. Have you ever been treated for reproductive tract conditions including HIV, herpes, gonorrhea, chlamydia, bacterial vaginosis, trichomoniasis, syphilis, or others? Yes No
   If "Yes", please circle above and/or specify "others" ___________________________________
9. Have you had vaginal intercourse **without** a condom in the **past 3 days**? Yes No
10. Have you received oral intercourse (contact between your vagina and your partner's tongue/mouth) in the **past 24 h**? Yes No
11. Have you received anal intercourse in the **past month**? Yes No
12. Do you use any vaginal products (douches, wipes, spermicides, lubricants) **regularly**? Yes No
    If "Yes", please specify what type(s): ____
13. Do you smoke or use tobacco products regularly (more than once a month)? Yes No
14. Do you have any concerns about your vaginal health today? ______________________________
    ____

[1] Personal data is collected in accordance with the National Institutes of Health regulations and policies regarding researching involving human subjects.

[2] These questions are designed to identify any factors which may affect the physical and chemical properties of the vaginal secretion sample you provide. Hormonal effects due to the natural menstrual cycle or contraceptive use, contact with semen or condom materials, or use of vaginal products may all alter the properties of cervicovaginal mucus.

Figure S1: Questionnaire used for all participants in this study.

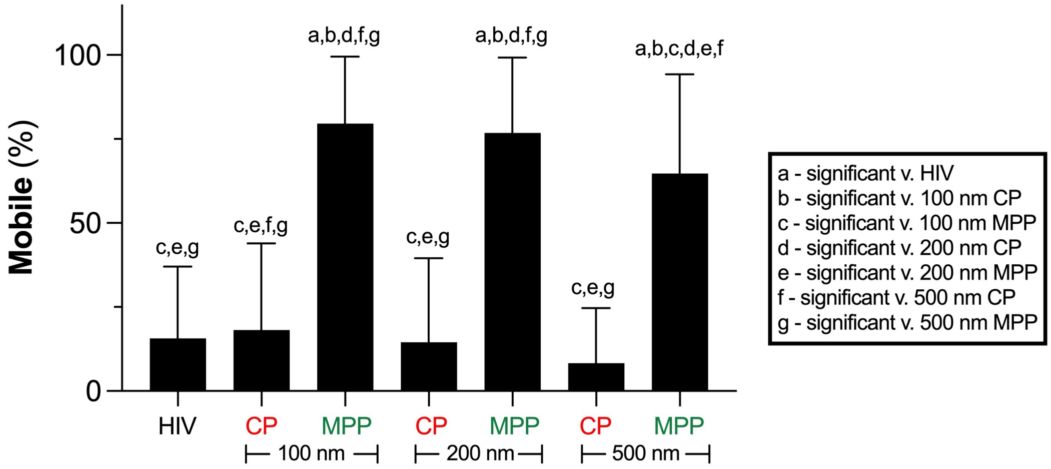

Figure S2: Estimated percentage of mobile particles at $\tau = 1$ s for all collected samples. Conventional particles (CP) were significantly less mobile than mucus penetrating particles (MPP). 500 nm CP were significantly less mobile than 100 nm CP (p=0.03). 500 nm MPP were significantly less mobile than 100 nm MPP (p<0.0001) and 200 nm MPP (p<0.0001). Significance was determined using one-way ANOVA in GraphPad Prism 8. Data are shown as mean ± SD. Significant differences are represented by letters above each dataset. Significance was determined using ANOVA with Tukey's multiple comparison test.

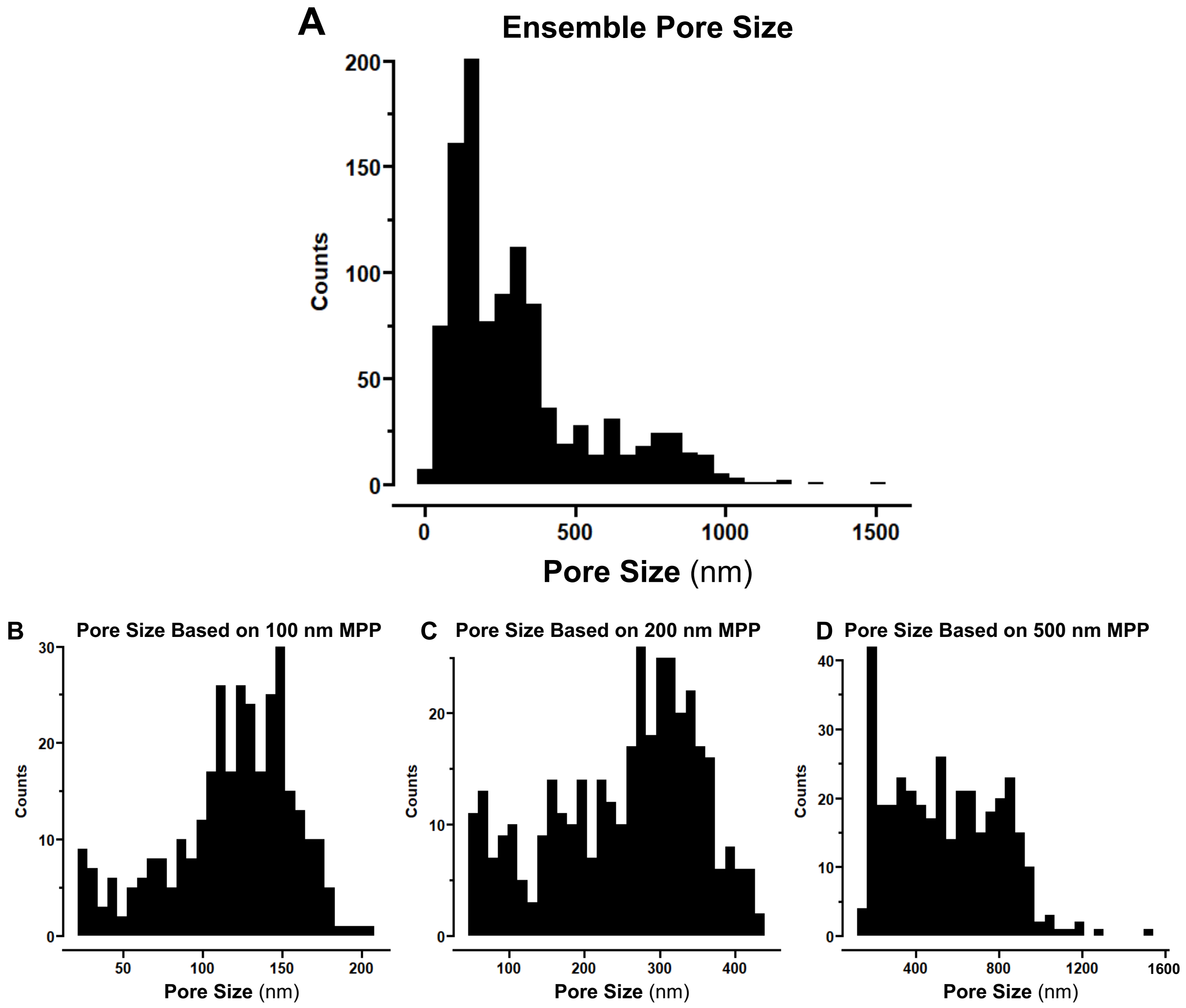

Figure S3: Histograms showing the pore size of CVM samples based on MSD calculations for (A) average of all tracked particles, (B) 100 nm MPPs, (C) 200 nm MPPs, (D) 500 nm MPPs.

Table S2: Pore size of CVM samples based on MSD calculations for average of all tracked particles, 100 nm MPPs, 200 nm MPPs, and 500 nm MPPs.

	**Mean Pore Size** (nm)	**Median Pore Size** (nm)	**Standard Deviation** (nm)	**Standard Error of Mean** (nm)
**Ensemble**	309.0	241.5	239.9	7.4
**100 nm**	118.3	124.1	38.7	2.1
**200 nm**	252.3	273.1	98.4	5.1
**500 nm**	542.8	529.9	259.7	13.7

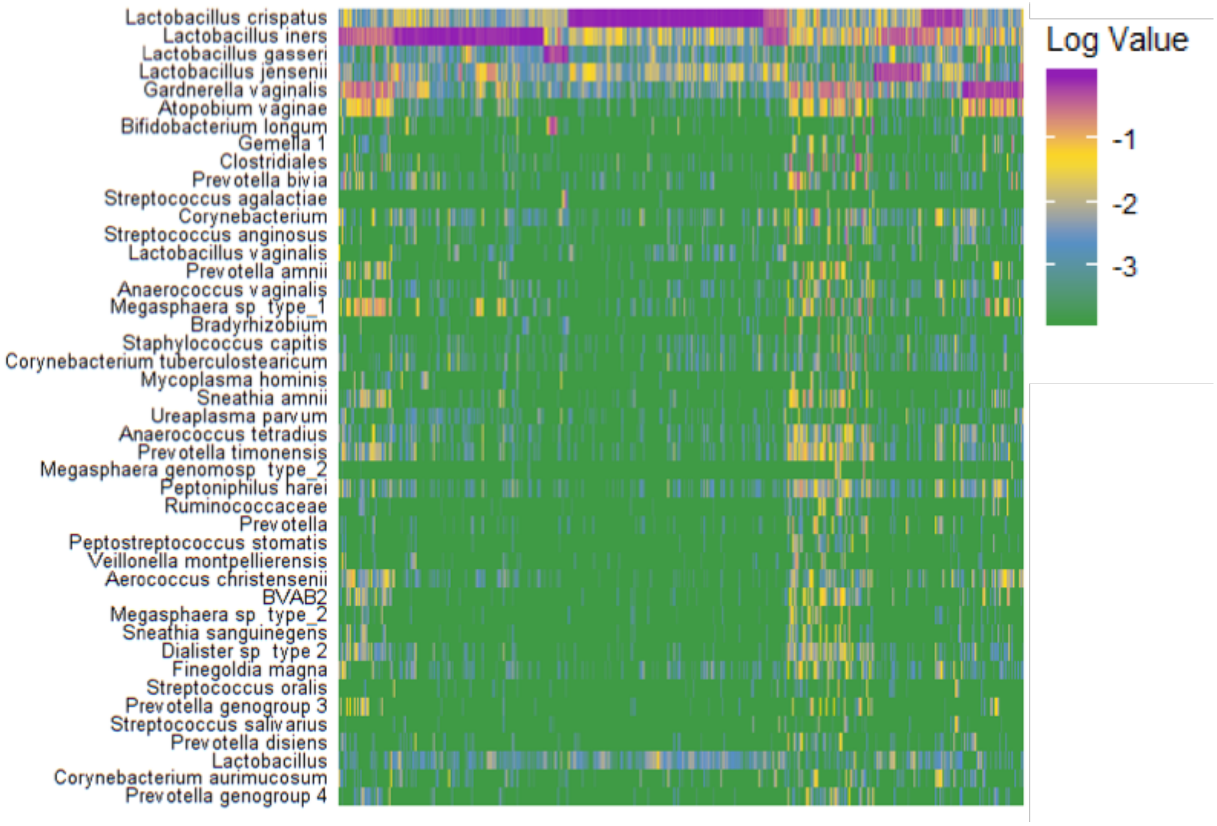

Figure S4: Heatmap showing vaginal bacteria presence in collected vaginal swab samples. Each row represents a bacterial species, as denoted by the row labels. Each column represents an individual samples. The intersecting cell is colored based on the bacterial presence, with purple indicating high levels of bacteria present, and green representing low levels.

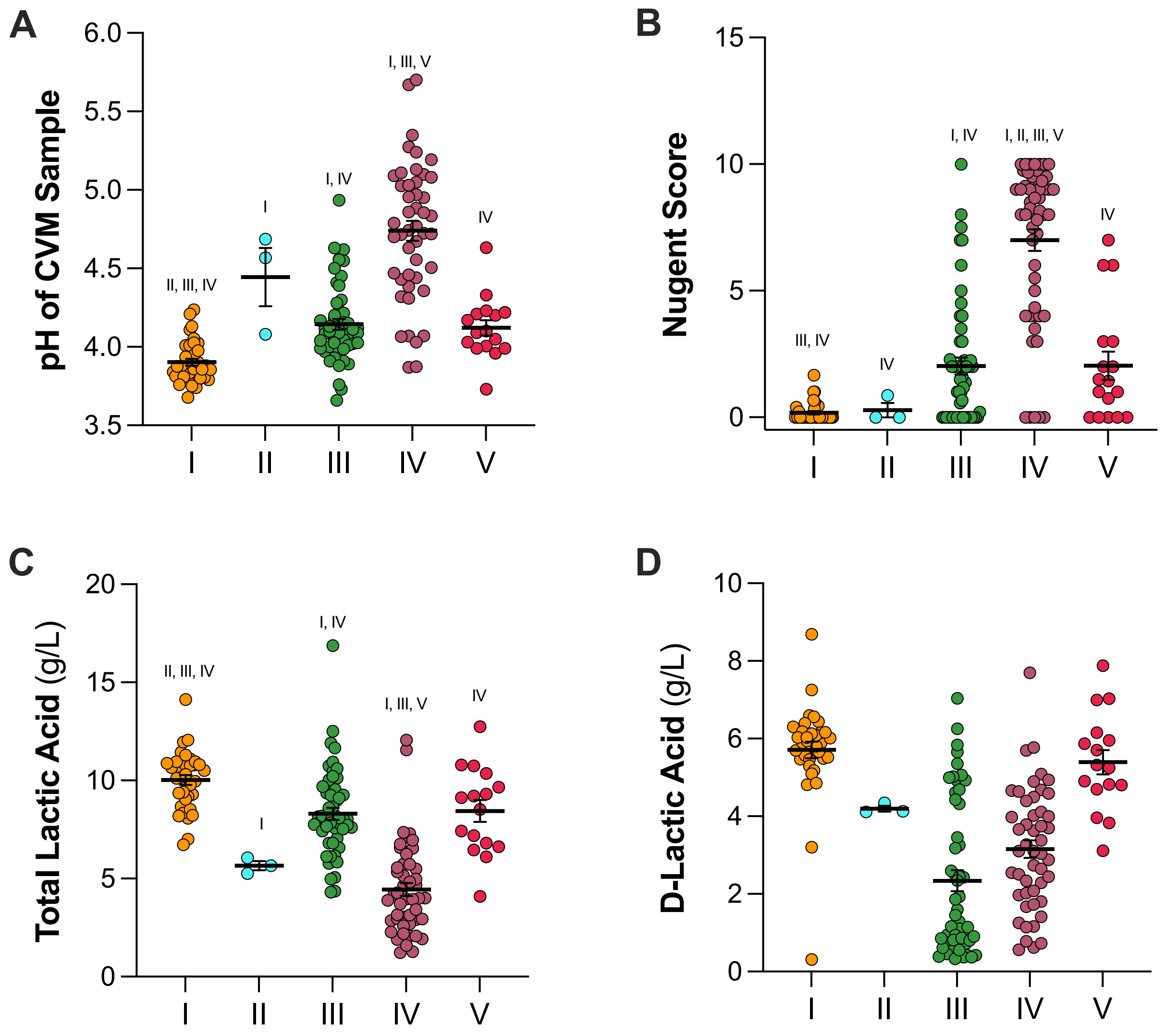

Figure S5: Sample characteristics based on vaginal microbiota CST. (A) pH, (B) Nugent score, (C) total lactic acid (g/L), (D) D-lactic acid (g/L) Data are shown as mean ± SD. Significant differences are represented by CST groups listed above each dataset. Significance was determined using ANOVA with Tukey's multiple comparison test.

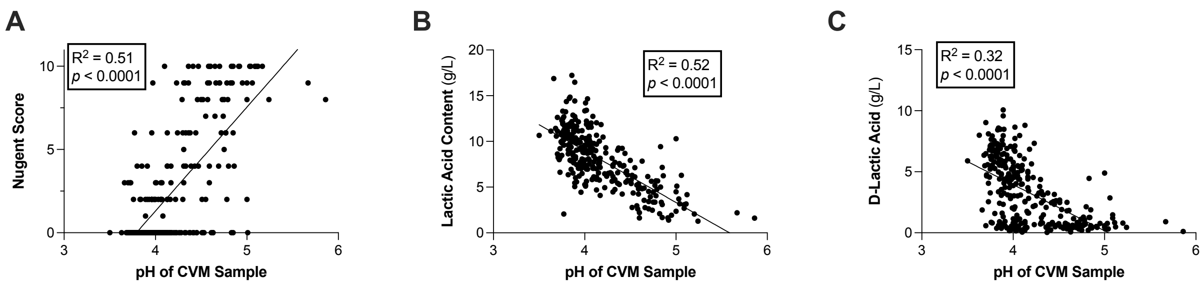

Figure S6: Cervicovaginal mucus (CVM) properties are strongly correlated with pH. (A) Nugent score significantly increases as pH increases. (B) Lactic acid content of CVM samples significantly decreases as pH increases. (C) Concentration of D-lactic acid significantly decreases as pH increases. Simple linear regressions are shown on each graph. R^2 and *p*-values were calculated using GraphPad Prism 8

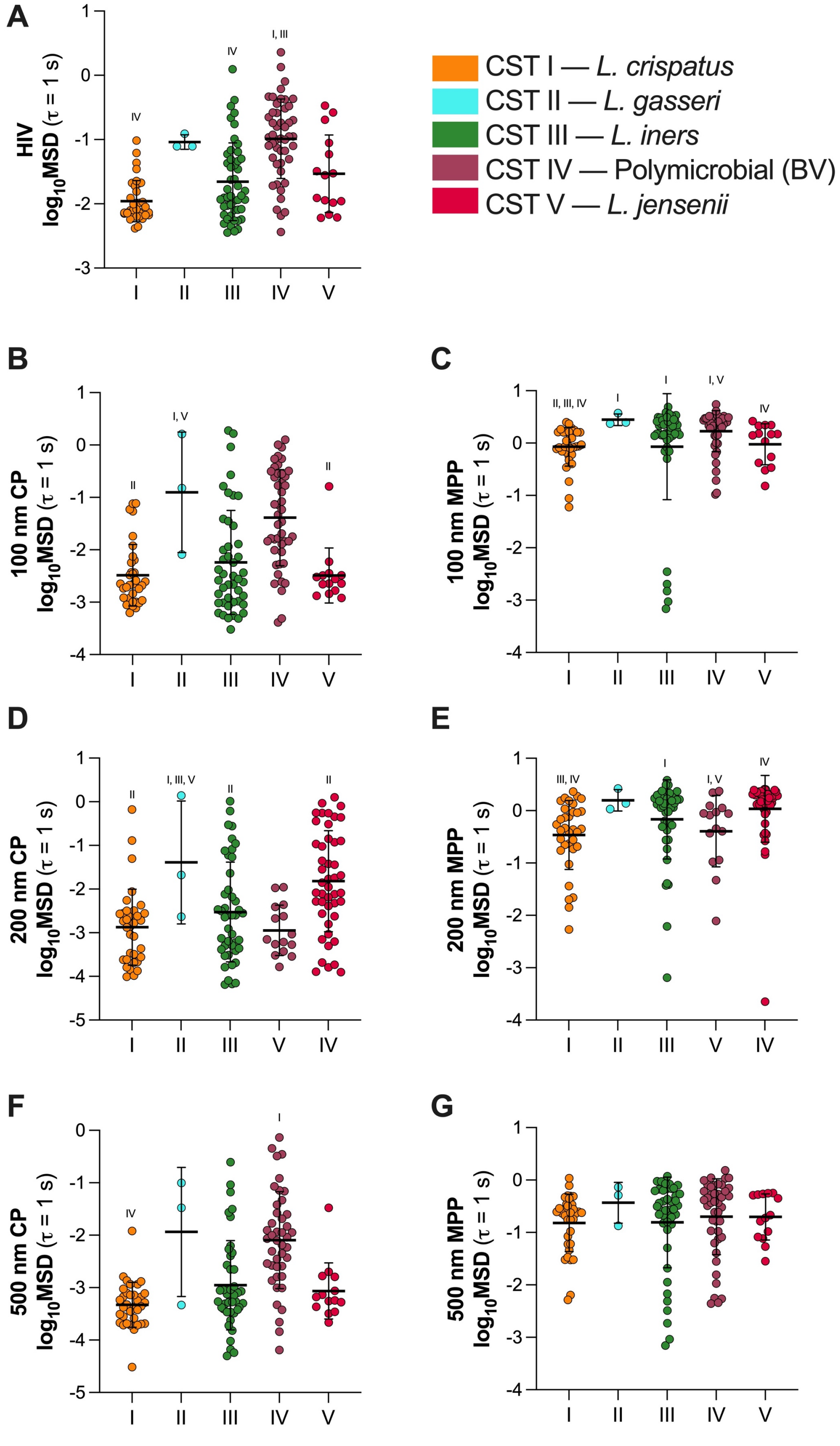

Figure S7: The $\log_{10}$(MSD) of each particle type at τ = 1 s within each CST. (A) HIV virion MSD, (B) 100 nm CP MSD, (C) 100 nm MPP MSD, (D) 200 nm CP MSD, (E) 200 nm MPP MSD, (F) 500 nm CP MSD, (G) 500 nm MPP MSD. Data are shown as mean ± SD. Significant differences are represented by CST groups listed above each dataset. Significance was determined using ANOVA with Tukey's multiple comparison test.

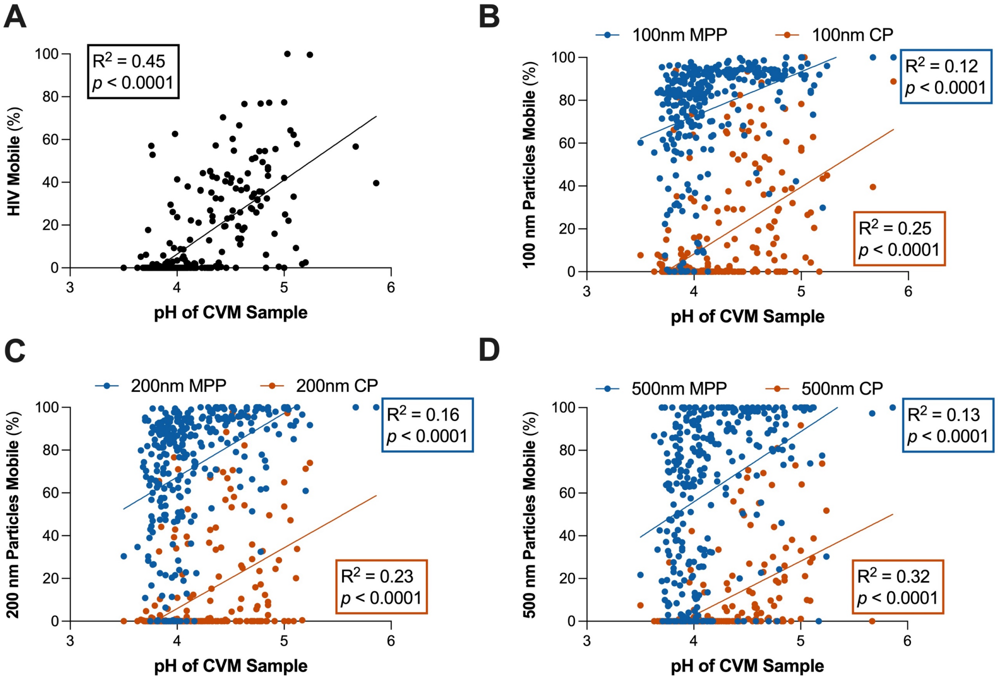

Figure S8: Cervicovaginal mucus (CVM) barrier properties are strongly correlated with pH. (A) HIV becomes significantly more mobile in CVM samples with an increased pH. (B) 100 nm MPP and 100 nm CP become significantly more mobile in CVM samples as pH increases. (C) 200 nm MPP and CP become significantly more mobile in CVM samples as pH increases. (D) 500 nm MPP and CP become significantly more mobile as pH increases. Mobility is calculated at τ = 1 s. Simple linear regressions are shown on each graph. R^2 and *p*-values were calculated using GraphPad Prism 8.

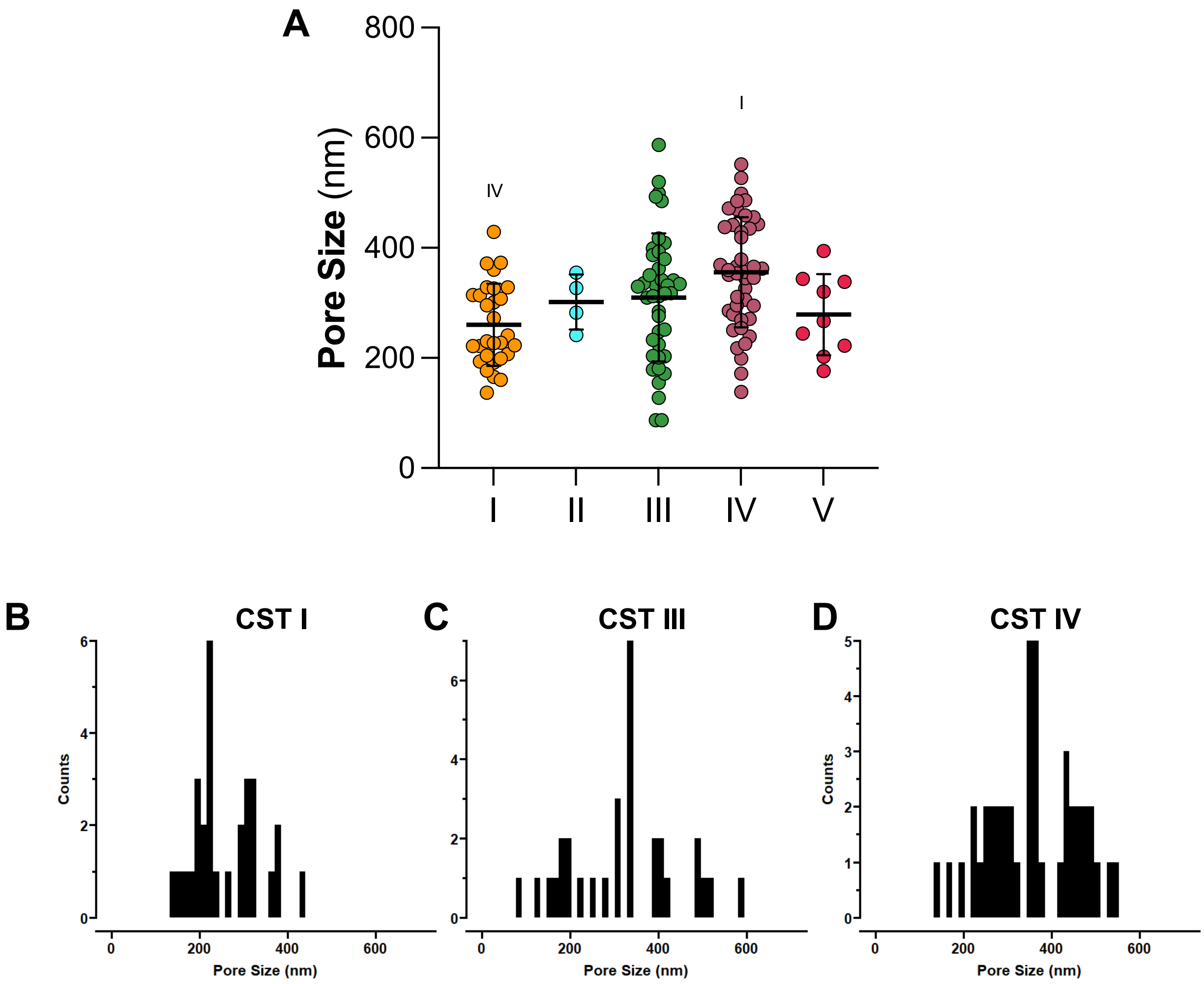

Figure S9: Pore size distributions for each CST. (A) Pore size comparison for each CST, where CST I had a significantly smaller pore size than CST IV (p = 0.0008). (B) Histogram of pore sizes within CST I samples. (C) Histogram of pore sizes within CST III samples. (D) Histogram of pore sizes within CST IV samples. Significance was determined using ANOVA with Kruskal-Wallis multiple comparison test.

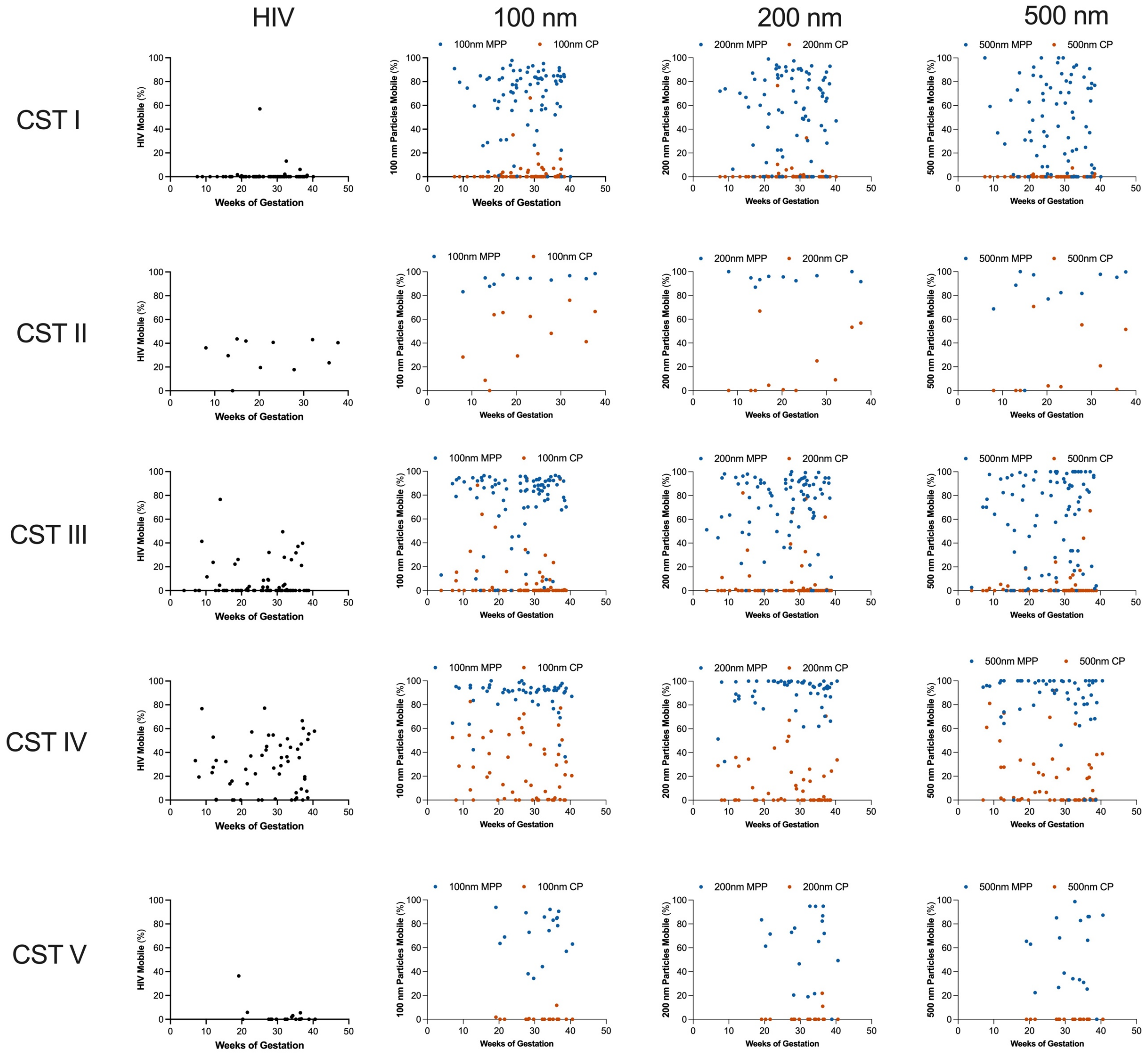

Figure S10: Changes in estimated percentage of mobile particles at $\tau = 1$ s over the course of pregnancy for HIV, 100 nm particles, 200 nm particles, and 500 nm particles where MPP are shown in green and CP are shown in red, broken down by sample CST.
